# Genes encoding neuropeptide receptors are epigenetic markers in patients with head and neck cancer: a site-specific analysis

**DOI:** 10.18632/oncotarget.19356

**Published:** 2017-07-18

**Authors:** Kiyoshi Misawa, Atsushi Imai, Daiki Mochizuki, Yuki Misawa, Shiori Endo, Seiji Hosokawa, Ryuji Ishikawa, Masato Mima, Kazuya Shinmura, Takeharu Kanazawa, Hiroyuki Mineta

**Affiliations:** ^1^ Department of Otolaryngology/Head and Neck Surgery, Hamamatsu University School of Medicine, Shizuoka, Japan; ^2^ Department of Tumour Pathology, Hamamatsu University School of Medicine, Shizuoka, Japan; ^3^ Department of Otolaryngology/Head and Neck Surgery, Jichi Medical University, Tochigi, Japan

**Keywords:** neuropeptide receptors, GPCR, head and neck cancer, epigenetic markers, metastases

## Abstract

Staging and pathological grading systems are useful but imperfect predictors of recurrence in head and neck squamous cell carcinoma (HNSCC). To identify potential prognostic markers, we examined the methylation status of eight neuropeptide receptor gene promoters in 231 head and neck squamous cell carcinomas. The *NPFFR1*, *NPFFR2*, *HCRTR1*, *HCRTR2*, *NPY1R*, *NPY2R*, *NPY4R*, and *NPY5R* promoters were methylated in 80.5%, 79.2%, 67.1%, 73.2%, 35.1%, 36.4%, 38.5%, and 35.9% of the samples, respectively. In a multivariate Cox proportional hazards analysis, the odds ratio for recurrence was 2.044 (95% confidence interval [CI], 1.323–3.156; P = 0.001) when the *NPY2R* promoter was methylated. In patients without lymph node metastasis (n = 100), methylation of *NPY2R* (compared with methylation of the other seven genes) best correlated with poor disease-free survival (DFS) (odds ratio, 2.492; 95% CI, 1.190–5.215; P = 0.015). In patients with oral cancer (n = 69), methylated *NPY1R* and *NPY2R* were independent prognostic factors for poor DFS, both individually and, even more so, in combination (odds ratio, 3.90; 95% CI, 1.523–9.991; P = 0.005). Similar findings were observed for *NPY2R* and *NPY4R* in patients with oropharyngeal cancer (n = 162) (odds ratio, 5.663; 95% CI, 1.507–21.28; P = 0.010).

## INTRODUCTION

Head and neck cancer is the eighth most common cancer worldwide with approximately 650,000 new cases reported annually [1]. More than 90% of head and neck cancers are squamous cell carcinomas; hence, the term “head and neck cancer” is often applied to all carcinomas that arise from the epithelium lining the sinonasal tract, oral cavity, pharynx, or larynx and show microscopic evidence of squamous differentiation [2]. The present standard management strategies include constructive and multimodal treatments such as surgery, radiotherapy, and chemotherapy. Despite these aggressive treatments, long-term survival rates are poor and remain between 40% and 50% [3]. Improvement of patient care requires molecular classification of head and neck squamous cell carcinomas (HNSCCs) that provides both prognostic and mechanistic information.

Technological advances revealed aberrant expression of G protein-coupled receptors (GPCRs) in human tumors owing to events such as gene point mutations, gene silencing via promoter methylation, and changes in gene copy number [4]. The GPCR family consists of six receptor groups with different pharmacological properties: rhodopsin-like GPCRs (Class A), secretin-like GPCRs (Class B), metabotropic glutamate receptors (Class C), fungal mating pheromone receptors (Class D), cAMP receptors (Class E), and frizzled/smoothened receptors (Class F) [5]. Class A, the largest and best-studied group, consists of four subgroups (α, β, γ, and δ) and includes several members that play a major part in tumor biology [6]. Although GPCRs regulate many aspects of tumorigenesis, only a few GPCR inhibitors are currently used to treat cancer. Potential targets for drug development include novel cancer-associated GPCRs identified via genome-wide analyses of several human tumor types [4].

Aberrant promoter methylation, a hallmark of cancer cells, accounts for the inactivation of many tumor suppressor genes. In HNSCC, methylation of gene promoters is a common mechanism of transcriptional silencing [7–9]. Notably, epigenetic repression of GPCR expression correlates with poor prognosis and the response to radiotherapy and chemotherapy [10].

The aim of this study was to determine the methylation status of eight GPCR-encoding genes in HNSCCs and its relationship to recurrence, survival, and clinical characteristics (e.g., tumor location and lymph node metastasis). All eight genes (*NPFFR1*, *NPFFR2*, *HCRTR1*, *HCRTR2*, *NPY1R*, *NPY2R*, *NPY4R*, and *NPY5R*) encode neuropeptide receptors and are in the Class A β subgroup. This study is the first to implicate neuropeptide receptors in the genesis of HNSCC.

## RESULTS

### Analysis of the methylation status and expression of neuropeptide receptor genes

**Figure 1:**
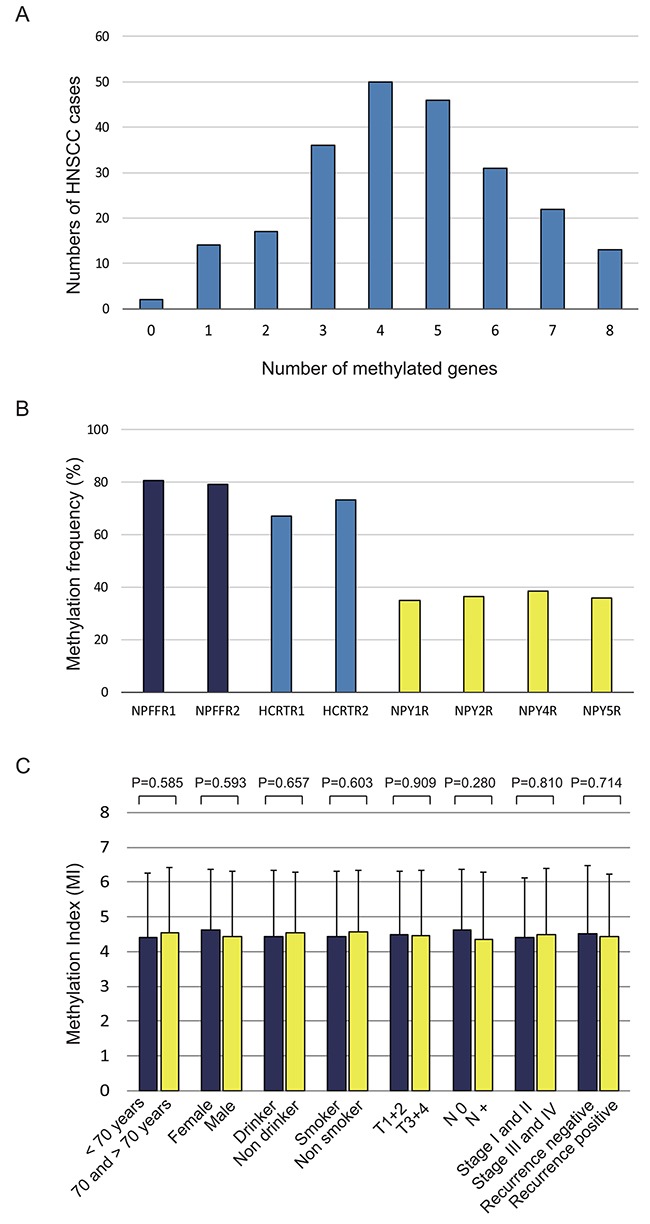
Methylation of the neuropeptide receptor gene promoters in 231 head and neck squamous cell carcinoma samples **(A)** Bar graph showing the percentage of tumors that express zero to eight of the methylated target genes. **(B)** Bar graph showing the methylation frequencies of the eight genes. **(C)** Bar graph showing the methylation indices (MIs) according to selected clinical parameters. The mean MI for each parameter was determined by using Student's t-test.

Quantitative methylation-specific polymerase chain reaction (PCR) was used to assess the promoter methylation status of eight genes encoding neuropeptide receptors in 231 primary HNSCC samples. At least one of these genes was methylated in almost all samples (229 of 231 samples, 99.1%). The mean number of methylated genes per sample was 4.46 (range, 0–8) (Figure 1A). The methylation rates for the eight genes were as follows: *NPFFR1*, 80.5%; *NPFFR2*, 79.2%; *HCRTR1*, 67.1%; *HCRTR2*, 73.2%; *NPY1R*, 35.1%; *NPY2R*, 36.4%; *NPY4R*, 38.5%; and *NPY5R*, 35.9% (Figure 1B, Supplementary Figure 1). Relative mRNA expression of the eight genes was assessed in 41 of the 231 tumor specimens via quantitative reverse transcription PCR (Supplementary Figure 2).

### Correlation between the methylation status of neuropeptide receptor gene promoters and clinicopathological parameters

The methylation index (MI) was defined as the ratio of the number of methylated genes and the number of tested genes in each sample. Continuous marker methylation analyses showed no association between the MI for any of the eight target genes and age at disease onset, sex, alcohol consumption, smoking status, tumor size, lymph node status, clinical stage, or recurrence (Figure 1C).

Associations between the methylation status of the target genes and the clinicopathological features of the patients are summarized in Table 1. Methylation of the *HCRTR2* promoter significantly correlated with lymph node metastasis (P = 0.040), methylation of the *NPY1R* promoter significantly correlated with smoking status (P = 0.041), and methylation of the *NPY2R* promoter significantly correlated with age (P = 0.040) and recurrence (P = 0.004).

**Table 1:** Distribution of methylation status by selected epidemiologic and clinical characteristics

				Characteristics					Age						Gender					Smoking status						Alcohol exposure			
Gene	Methylation status			Overall(%)					< 70		> 70		P ^†^		Female		Male	P ^†^		Smoker		Non smoker		P ^†^		Drinker		Non drinker	P ^†^
NPFFR1	Yes			186(80.5)					119		67				29		157			140		46				136		50	
	No			45(19.5)					30		15		0.735		7		38	0.995		35		10		0.725		33		12	0.977
NPFFR2	Yes			183(79.2)					118		65				31		152			135		48				134		49	
	No			48(20.8)					31		17		0.989		5		43	0.267		40		8		0.169		35		13	0.966
HCRTR1	Yes			155(67.1)					103		52				26		129			120		35				114		41	
	No			76(33.9)					46		30		0.377		10		66	0.476		55		21		0.4		55		21	0.849
HCRTR2	Yes			169(73.2)					108		61				27		142			126		43				120		49	
	No			62(27.8)					41		21		0.754		9		53	0.786		49		13		0.482		49		13	0.223
NPY1R	Yes			81(35.1)					48		33				14		67			55		26				25		56	
	No			150(64.9)					101		49		0.221		22		128	0.601		120		30		0.041*		37		113	0.31
NPY2R	Yes			84(36.4)					47		37				13		71			64		20				61		23	
	No			147(63.6)					102		45		0.04*		23		124	0.973		111		36		0.908		108		39	0.888
NPY4R	Yes			89(38.5)					55		34				12		77			73		16				70		19	
	No			142(61.5)					94		48		0.496		24		118	0.486		102		40		0.079		99		43	0.136
NPY5R	Yes			83(35.9)					59		24				14		69			61		22				57		26	
	No			148(64.1)					90		58		0.117		22		126	0.687		114		34		0.548		112		36	0.249

	Tumor size				Lympho-node status			Stage						HPV status							Recurrence events					
Gene	T1-2	T3-4	P ^†^		N0	N+	P ^†^	I, II		III, IV		P †		positive		negative			P †		positive		negative		P ^†^	
NPFFR1	89	97			83	103		50		136				32		154					63		123			
	23	22	0.694		17	28	0.406	11		34		0.739		5		38			0.492		22		23		0.061	
NPFFR2	91	92			82	101		51		132				29		154					67		116			
	21	27	0.461		18	30	0.363	10		38		0.325		8		38			1		18		30		0.91	
HCRTR1	73	82			71	84		40		115				26		129					57		98			
	39	37	0.547		29	47	0.27	21		55		0.767		11		63			0.848		28		48		0.992	
HCRTR2	85	84			80	89		48		121				27		142					56		113			
	27	35	0.363		20	42	0.04*	13		49		0.256		10		50			1		29		33		0.057	
NPY1R	40	41			36	45		18		63				22		59					35		46			
	72	78	0.841		64	86	0.795	43		107		0.289		15		133			0.001*		50		100		0.137	
NPY2R	36	48			38	46		20		64				13		71					41		43			
	76	71	0.196		62	85	0.651	41		106		0.498		24		121			1		44		103		0.004*	
NPY4R	43	46			37	52		19		70				14		75					32		57			
	69	73	0.967		63	79	0.677	42		100		0.167		23		117			1		53		89		0.834	
NPY5R	44	39			34	49		23		60				14		67					33		50			
	68	80	0.303		66	82	0.593	38		110		0.736		23		125			1		52		96		0.484	

† Chi-squared test.

* P<0.05.

### Kaplan-Meier analysis

**Figure 2:**
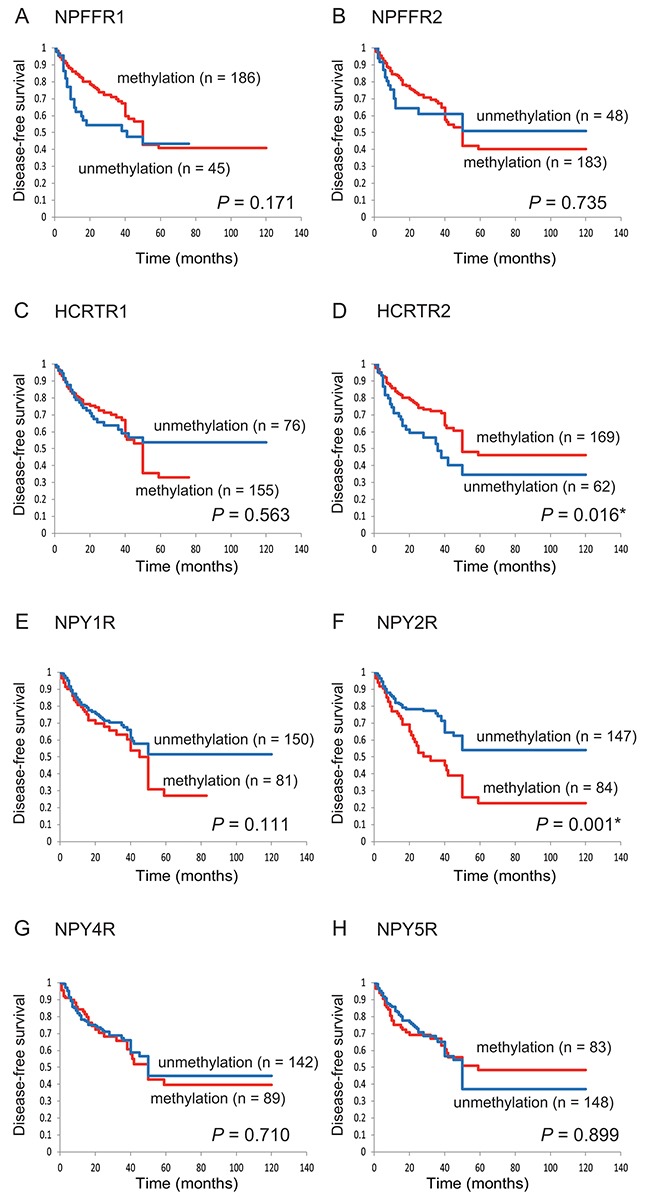
Kaplan-Meier survival curves for the 231 patients with head and neck squamous cell carcinoma according to the methylation status of the eight target genes Disease-free survival for **(A)** NPFFR1, **(B)** NPFFR2, **(C)** HCRTR1, **(D)** HCRTR2, **(E)** NPY1R, **(F)** NPY2R, **(G)** NPY4R and **(H)** NPY5R. The log-rank test was used to compare the survival times in patients with methylated (red lines) and unmethylated (blue lines) genes.

The Kaplan-Meier survival curves for each of the eight target genes in all patients are shown in Figure 2. Disease-free survival (DFS) time did not differ significantly in patients with methylated versus unmethylated genes, with two notable exceptions: it was significantly shorter when *HCRTR2* was unmethylated (P = 0.016) and when *NPY2R* was methylated (P = 0.001). Additional analysis of only the patients without lymph node metastasis (n = 100) revealed shorter DFS times for methylated versus unmethylated *NPY2R* (P = 0.026), but no differences for the other seven genes (Supplementary Figure 3).

### Prognostic value of the methylation status of neuropeptide receptor gene promoters

The association between methylation and risk of recurrence was estimated via multivariate analysis using a Cox proportional hazards model adjusted for age, sex, smoking status, alcohol consumption, and clinical stage. In patients in whom the *NPY2R* promoter was methylated (n = 84), the adjusted odds ratio for recurrence was 2.044 (95% confidence interval [CI], 1.323–3.156; P = 0.001) (Table 2). Notably, the odds ratio was significantly higher in patients with no lymph node metastasis (n = 100) in whom the *NPY2R* promoter was methylated versus unmethylated (odds ratio, 2.492; 95% CI, 1.190–5.215; P = 0.015) (Table 3). There was no association between the methylation status of the *HCRTR2* promoter and recurrence regardless of lymph node status (Tables 2, 3). Remarkably, we found that the mode of therapy, HPV status, smoking status, alcohol intake, tumor stage, and gene methylation status all indicated the likelihood of recurrence in patients with methylated *NPY1R* and *NPY2R* promoters. We have included these data in the multivariate analysis in Supplementary Table 1.

**Table 2:** Methylation status of individual genes and associations with disease-free survival using Cox proportional hazards model in 231 patients

Gene	Methylation status	Overall (%)	Recurrence events		Adjusted HR (95% CI) †
			Positive (N = 88)	Negative (N = 143)	
NPFFR1	Yes	186(80.5)	66	120	
	No	45(19.5)	22	23	0.693 (0.424-1.133)
NPFFR2	Yes	183(79.2)	69	114	
	No	48(20.8)	19	29	0.915 (0.540-1.550)
HCRTR1	Yes	155(67.1)	58	97	
	No	76(33.9)	30	46	1.126 (0.708-1.791)
HCRTR2	Yes	169(73.2)	55	114	
	No	62(27.8)	33	29	0.582 (0.320-1.059)
NPY1R	Yes	81(35.1)	32	49	
	No	150(64.9)	56	94	1.407 (0.910-2.176)
NPY2R	Yes	84(36.4)	42	42	
	No	147(63.6)	46	101	2.044 (1.323-3.156)*
NPY4R	Yes	89(38.5)	33	56	
	No	142(61.5)	55	87	1.033 (0.660-1.617)
NPY5R	Yes	83(35.9)	32	51	
	No	148(64.1)	56	92	1.006 (0.646-1.567)

† Adjusted for age, gender, smoking status, alcohol exposure and stage.

* P<0.05.

**Table 3:** Methylation status of individual genes and associations with disease-free survival using Cox proportional hazards model in 100 patients with N0 lymph-node status

Gene	Methylation status	Overall (%)	Recurrence events		Adjusted HR (95% CI) †
			Positive (N = 30)	Negative (N = 70)	
NPFFR1	Yes	83(83.0)	23	60	
	No	17(17.0)	7	10	0.831 (0.348-1.981)
NPFFR2	Yes	82(82.0)	23	59	
	No	18(18.0)	7	11	0.656 (0.266-1.617)
HCRTR1	Yes	71(71.0)	22	49	
	No	29(29.0)	8	21	1.162 (0.469-2.879)
HCRTR2	Yes	80(80.0)	22	58	
	No	20(20.0)	8	12	0.465 (0.192-1.131)
NPY1R	Yes	36(36.0)	14	22	
	No	64(64.0)	16	48	1.568 (0.736-3.342)
NPY2R	Yes	38(38.0)	16	22	
	No	62(62.0)	14	48	2.492 (1.190-5.215)*
NPY4R	Yes	37(37.0)	12	25	
	No	63(63.0)	18	45	0.926 (0.423-2.030)
NPY5R	Yes	34(34.0)	12	22	
	No	66(66.0)	18	48	1.118 (0.523-2.387)

† Adjusted for age, gender, smoking status, alcohol exposure and stage.

* P<0.05.

**Figure 3:**
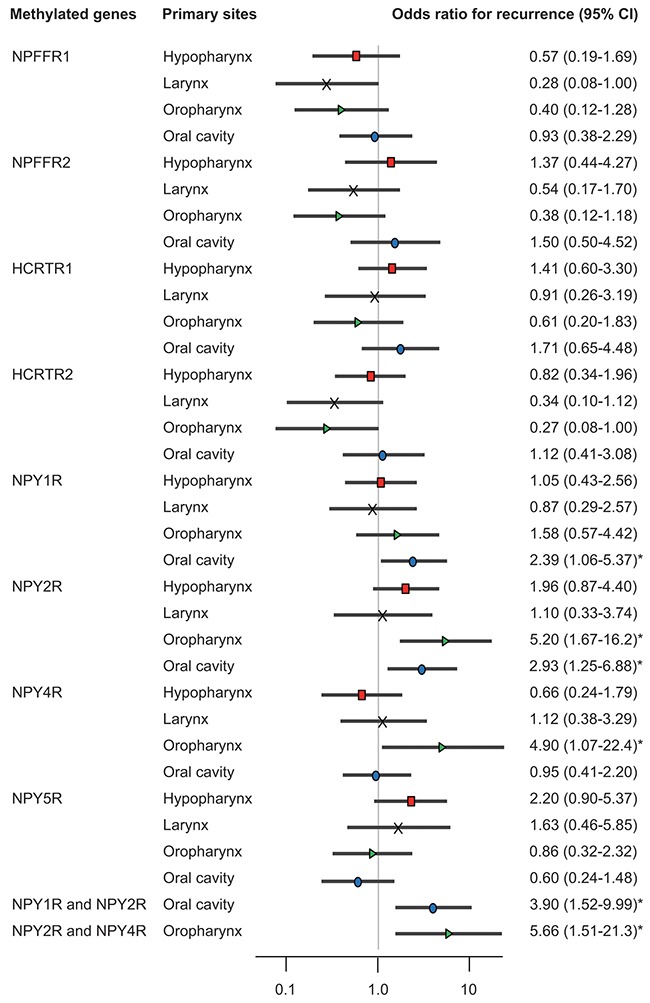
Risk of recurrence based on gene methylation in tumors with different origins Odds ratios for recurrence were determined by using a Cox proportional hazards model adjusted for age (≥70 versus <70 years), sex, smoking status, alcohol intake, and stage (I–III versus IV). CI: confidence interval.

Odds ratios for recurrence according to tumor origin were also determined. When the *NPY1R* and*NPY2R* promoters were methylated in patients with oral cancers, the ratios were 2.39 (95% CI, 1.06–5.37; P = 0.036) and 2.93 (95% CI, 1.25–6.88; P = 0.014), respectively, and the hazard rate was 3.9 times higher (95% CI, 1.52–9.99; P = 0.005) (Figure 3). Methylation of the *NPY2R* and *NPY4R* promoters correlated positively with recurrence in patients with oropharyngeal cancers, both individually (odds ratio, 5.20; 95% CI, 1.67–16.2; P = 0.005 and odds ratio, 4.90; 95% CI, 1.07–22.4; P = 0.004, respectively) and together (odds ratio, 5.66; 95 % CI, 1.51–21.3; P = 0.010).

### External validation of our results using data from The Cancer Genome Atlas (TCGA) database

The methylation status of neuropeptide receptor gene promoters was determined in an additional 516 HNSCC samples and 50 normal samples. The average β values for promoter methylation were significantly higher in the HNSCC samples than in the normal samples (P < 0.005) (Supplementary Figure 4). mRNA expression data were available for 497 HNSCC samples and 20 normal samples. *NPFFR2, HCRTR2, NPY1R, NPY2R*, and *NPY5R* promoter methylation correlated inversely with their respective mRNA levels in both the HNSCC and normal tissue samples (Supplementary Figure 5). To validate the prognostic implications of neuropeptide receptor gene methylation, we examined the data for the 386 HNSCC patients in TCGA database. DFS time was significantly longer in patients with an unmethylated *HCRTR1* promoter than in those with a methylated *HCRTR1* promoter (P = 0.038) (Supplementary Figure 6).

## DISCUSSION

Identifying epigenetic modifications of the *NPFFR*, *HCRTR*, and *NPYR* genes is important for understanding how tumors arise and whether they will recur. Using real-time PCR, we examined the methylation status of these genes, all of which encode G protein-coupled neuropeptide receptors, in 231 HNSCCs originating in the hypopharynx, larynx, oropharynx, or oral cavity. We also compared the methylation status of matched HNSCC and normal samples using data from TCGA database. We found that aberrant methylation of the *NPY1R*, *NPY2R*, and *NPY4R* promoters correlated positively with recurrence in patients with HNSCC.

Members of the GPCR family include the neuropeptide FF (NPFF) receptors, of which there are two subtypes, NPFFR1 and NPFFR2, which bind the RF-amide related peptides and FF neuropeptides, respectively [11]. NPFFR1 and NPFFR2 are about 50% identical and are closely related to neuropeptide Y receptors and orexin receptors (30–35% homology) [12]. Classically, the actions of orexins are mediated by two receptors, orexin receptor type 1 and type 2, which are encoded by the *HCRTR1* and *HCRTR2* genes, respectively. Orexins transiently increase intracellular calcium levels through Gq-dependent and -independent pathways [13] and markedly inhibit cell proliferation in various cancer cell lines by inducing apoptosis [14]. In a previous study, primary colorectal tumors and hepatic metastases expressed *HCRTR1* mRNA regardless of their location or Dukes stage, whereas adjacent normal colonocytes did not express *HCTRTR1* mRNA [15]. Loss of expression of *HCRTR2* correlates with hypermethylation of *HCRTR2* in endometrial cancer compared with normal endometrium [16].

Neuropeptide Y (NPY) activates five GPCRs, namely, NPY1R, NPY2R, NPY4R, NPY5R, and NPY6R [17]. It is one of the most abundantly distributed neurotransmitters and vasoconstrictors in the central and peripheral nervous system. Although the *NPY6R* gene is functional in rabbits and mice, it is absent in rats and considered a pseudogene in primates and pigs [18]. NPY regulates food intake, blood pressure, and circadian rhythms, as well as other physiological activities [19]. Via NPY1R, NPY inhibits the growth of hepatocellular carcinomas by inactivating the mitogen-activated protein kinase signaling pathway [20]. Significant associations between cumulative arsenic exposure and the methylation level of the *NPY2R* gene have been observed in smoking-unrelated urothelial carcinomas [21]. NPY2R is often strongly expressed in neuroblastomas, paragangliomas, and renal carcinomas [22], and NPY2R agonists such as BIM-43004-1 suppress the growth of human pancreatic cancer xenografts in mice [23]. NPY4R is expressed in peripheral organs including the gastrointestinal tract, liver, pancreas, and heart [24]. The specific NPY4R agonist, BA-129 inhibits the proliferation of pancreatic cancer cells *in vitro* [25], and genetic and structural variations in NPY4R have been implicated in the pathogenesis of obesity [26].

Type 1 and 2 galanin receptors, type 1 tachykinin receptors, and type 1 somatostatin receptors, which are also neuropeptide GPCRs, are encoded by the *GALR1*, *GALR2*, *TACR1*, and *SSTR1* genes, respectively, and the methylation frequencies of these genes are 51.0%, 37.6%, 34.0%, and 64.0%, respectively [27–29]. In oral cancers, *GALR1* promoter hypermethylation significantly and inversely correlates with DFS time [29]. In oropharyngeal cancers, the odds ratio for recurrence is higher when the *GALR2* promoter is methylated versus unmethylated [29]. On the other hand, there is no association between *TACR1* or *SSTR1* methylation and prognosis in HNSCC patients regardless of tumor origin [27, 28].

Our study associates *NPY1R*, *NPY2R,* and *NPY4R* methylation with tumor recurrence in oral and oropharyngeal cancers. This finding may facilitate HNSCC screening and the development of surveillance algorithms. Simultaneous analysis of the methylation status of multiple neuropeptide GPCR-encoding genes will allow us to better predict tumor-related events, assess biological behavior, and design targeted therapies for HNSCCs.

GPCRs and their signal transduction networks affect physiological, immunological, and endocrinological processes and stem cell biology [4]. This is the first study to show epigenetic regulation of eight neuropeptide receptor genes in HNSCC. It addresses the significant challenges unique to the identification of GPCR biomarkers and, as a GPCR-targeted study, may facilitate the identification of drugs for cancer prevention and treatment [30]. Both GPCRs and receptor-tyrosine kinases (RTKs) regulate extensive signaling networks, control multiple cell functions, and participate in many diseases including cancer [31]. Transactivation of epidermal growth factors, which are RTKs, by GPCRs has been reported; hence, specific disruption of the crosstalk between these receptor types, even without inhibition of their activities, should substantially impede disease progression [32].

Studies involving human specimens and high-throughput profiling platforms may be susceptible to measurement bias from a variety of sources. The present study suggests that the methylation status of the *NPY1R*, *NPY2R*, and *NPY4R* genes is an independent indicator of DFS in patients with oral and/or oropharyngeal cancers. Our findings support the use of methylation markers in patient selection for adjuvant therapy after initial surgical treatment and may aid oropharyngeal cancer screening and surveillance programs. However, they are preliminary and hence need to be validated in larger and more homogeneous HNSCC patient cohorts.

## MATERIALS AND METHODS

### Tumor samples

Two hundred and thirty-one primary HNSCC samples were obtained from patients during surgery at the Department of Otolaryngology, Hamamatsu University School of Medicine. All patients provided written informed consent, and the study protocol was approved by the Institutional Review Board of the Hamamatsu University School of Medicine. Pertinent information including age, sex, smoking status, alcohol consumption, lymph node status, tumor site, tumor size, and clinical stage was obtained from the patients’ medical records (Supplementary Table 2). The male:female ratio in the patient cohort was 195:36. The mean age was 65.4 years (range, 32–93). Primary tumors were located in the hypopharynx (n = 59), larynx (n = 45), oropharynx (n = 58), or oral cavity (n = 69).

### Quantitative methylation-specific PCR analysis

Extraction and bisulfite conversion of genomic DNA from 231 primary HNSCC and 36 noncancerous mucosal samples were performed using a MethylEasy Xceed Rapid DNA Bisulfite Modification Kit (TaKaRa, Tokyo, Japan) [33]. The methylation levels of the CpG islands in the promoters of the target genes were determined via quantitative methylation-specific PCR using the TaKaRa Thermal Cycler Dice TM Real Time System TP800 (TaKaRa). The primer sequences are listed in Supplementary Table 3. A standard curve was constructed by plotting known concentrations of serially diluted EpiScope^TM^ Methylated HeLa gDNA (TaKaRa). The normalized methylation value (NMV) was determined as follows: NMV = (Target gene-S/Target gene-FM)/(ACTB-S/ACTB-FM), where Target gene-S and Target gene-FM represent target gene methylation levels in the tumor sample and universal methylated DNA control, respectively, and ACTB-S and ACTB-FM represent *ACTB* (which encodes β-actin) methylation levels in the sample and control, respectively. Analysis was performed using the software (version 1.03A) for the Thermal Cycler Dice Real Time System TP800 according to the manufacturer's directions [34].

### RNA extraction and quantitative reverse transcription (qRT-)PCR

Total RNA was isolated with RNeasy Plus Mini kit (Qiagen, Valencia, CA, USA), and cDNA was synthesized with ReverTra Ace qPCR RT kit (Toyobo, Osaka, Japan). Primer sequences are shown in Supplementary Table 4. Target mRNA expression was compared between samples by normalization to *glyceraldehyde 3-phosphate dehydrogenase (GAPDH)* mRNA expression.

### Collection of publicly available data from TCGA

Aberrant DNA methylation data contained in TCGA (available in May 2017) were collected from the MethHC (http://methhc.mbc.nctu.edu.tw/php/index.php) [35] and cBioPortal (http://www.cbioportal.org/) databases [36] using the Infinium HumanMethylation450 platform (Illumina, Inc., San Diego, CA, USA) and are expressed as β values. The β value is a number between 0 (not methylated) and 1 (completely methylated) that represents the ratio of methylated allele intensity and overall intensity.

### Statistical analysis

Receiver-operator characteristic (ROC) curve analysis was performed using the NMVs for 36 HNSCC and 36 adjacent normal mucosal samples and the Stata/SE 13.0 system (Stata Corporation, TX, USA). The area under the ROC curve indicated the optimal sensitivity and specificity cutoff levels for distinguishing between the methylation levels in normal and HNSCC tissue, and the NMV thresholds were calculated for each target gene. The cutoff values were used to determine the methylation frequencies of the target genes (Supplementary Figure 1). The overall methylation rates in the individual samples were determined by calculating the MIs [37, 38].

Associations between the variables were assessed by using Student's t-test. Disease-free survival (DFS) was measured from the date of the initial treatment to the date of diagnosis of locoregional recurrence or distant metastasis. The Kaplan-Meier test was used to calculate survival probabilities, and the log-rank test was used to compare survival rates. The prognostic value of the methylation status was assessed by performing a multivariate Cox proportional hazards analysis adjusted for age (≥70 versus <70 years), sex, alcohol intake, smoking status, and tumor stage (I–III versus IV) [39]. Differences with P < 0.05 were considered significant. All statistical analyses were performed by using StatMate IV software (ATMS Co., Ltd., Tokyo, Japan).

## SUPPLEMENTARY MATERIALS FIGURES AND TABLES









## References

[1] Parkin DM, Bray F, Ferlay J, Pisani P (2005). Global cancer statistics, 2002. CA Cancer J Clin.

[2] Pai SI, Westra WH (2009). Molecular pathology of head and neck cancer: implications for diagnosis, prognosis, and treatment. Annu Rev Pathol.

[3] Kanazawa T, Misawa K, Misawa Y, Uehara T, Fukushima H, Kusaka G, Maruta M, Carey TE (2015). G-protein-coupled receptors: next generation therapeutic targets in head and neck cancer?. Toxins (Basel).

[4] Bar-Shavit R, Maoz M, Kancharla A, Nag JK, Agranovich D, Grisaru-Granovsky S, Uziely B (2016). G protein-coupled receptors in cancer. Int J Mol Sci.

[5] Munk C, Isberg V, Mordalski S, Harpsoe K, Rataj K, Hauser AS, Kolb P, Bojarski AJ, Vriend G, Gloriam DE (2016). GPCRdb: the G protein-coupled receptor database - an introduction. Br J Pharmacol.

[6] Isberg V, Mordalski S, Munk C, Rataj K, Harpsoe K, Hauser AS, Vroling B, Bojarski AJ, Vriend G, Gloriam DE (2016). GPCRdb: an information system for G protein-coupled receptors. Nucleic Acids Res.

[7] Kang H, Kiess A, Chung CH (2015). Emerging biomarkers in head and neck cancer in the era of genomics. Nat Rev Clin Oncol.

[8] Arantes LM, de Carvalho AC, Melendez ME, Carvalho AL, Goloni-Bertollo EM (2014). Methylation as a biomarker for head and neck cancer. Oral Oncol.

[9] Meng RW, Li YC, Chen X, Huang YX, Shi H, Du DD, Niu X, Lu C, Lu MX (2016). Aberrant methylation of RASSF1A closely associated with HNSCC, a meta-analysis. Sci Rep.

[10] Kanazawa T, Misawa K, Carey TE (2010). Galanin receptor subtypes 1 and 2 as therapeutic targets in head and neck squamous cell carcinoma. Expert Opin Ther Targets.

[11] Ayachi S, Simonin F (2014). Involvement of mammalian RF-amide peptides and their receptors in the modulation of nociception in rodents. Front Endocrinol (Lausanne).

[12] Zajac JM (2001). Neuropeptide FF: new molecular insights. Trends Pharmacol Sci.

[13] Rouet-Benzineb P, Rouyer-Fessard C, Jarry A, Avondo V, Pouzet C, Yanagisawa M, Laboisse C, Laburthe M, Voisin T (2004). Orexins acting at native OX(1) receptor in colon cancer and neuroblastoma cells or at recombinant OX(1) receptor suppress cell growth by inducing apoptosis. J Biol Chem.

[14] Xu TR, Yang Y, Ward R, Gao L, Liu Y (2013). Orexin receptors: multi-functional therapeutic targets for sleeping disorders, eating disorders, drug addiction, cancers and other physiological disorders. Cell Signal.

[15] Voisin T, El Firar A, Fasseu M, Rouyer-Fessard C, Descatoire V, Walker F, Paradis V, Bedossa P, Henin D, Lehy T, Laburthe M (2011). Aberrant expression of OX1 receptors for orexins in colon cancers and liver metastases: an openable gate to apoptosis. Cancer Res.

[16] Dehan P, Canon C, Trooskens G, Rehli M, Munaut C, Van Criekinge W, Delvenne P (2013). Expression of type 2 orexin receptor in human endometrium and its epigenetic silencing in endometrial cancer. J Clin Endocrinol Metab.

[17] Ejaz A, LoGerfo FW, Khabbaz K, Pradhan L (2011). Expression of Neuropeptide Y, Substance P, and their receptors in the right atrium of diabetic patients. Clin Transl Sci.

[18] Matsumoto M, Nomura T, Momose K, Ikeda Y, Kondou Y, Akiho H, Togami J, Kimura Y, Okada M, Yamaguchi T (1996). Inactivation of a novel neuropeptide Y/peptide YY receptor gene in primate species. J Biol Chem.

[19] Gehlert DR (1998). Multiple receptors for the pancreatic polypeptide (PP-fold) family: physiological implications. Proc Soc Exp Biol Med.

[20] Lv X, Zhao F, Huo X, Tang W, Hu B, Gong X, Yang J, Shen Q, Qin W (2016). Neuropeptide Y1 receptor inhibits cell growth through inactivating mitogen-activated protein kinase signal pathway in human hepatocellular carcinoma. Med Oncol.

[21] Yang TY, Hsu LI, Chiu AW, Pu YS, Wang SH, Liao YT, Wu MM, Wang YH, Chang CH, Lee TC, Chen CJ (2014). Comparison of genome-wide DNA methylation in urothelial carcinomas of patients with and without arsenic exposure. Environ Res.

[22] Korner M, Reubi JC (2007). NPY receptors in human cancer: a review of current knowledge. Peptides.

[23] Liu CD, Slice LW, Balasubramaniam A, Walsh JH, Newton TR, Saxton RE, McFadden DW (1995). Y2 receptors decrease human pancreatic cancer growth and intracellular cyclic adenosine monophosphate levels. Surgery.

[24] Hausman GJ, Barb CR, Dean RG (2008). Patterns of gene expression in pig adipose tissue: insulin-like growth factor system proteins, neuropeptide Y (NPY), NPY receptors, neurotrophic factors and other secreted factors. Domest Anim Endocrinol.

[25] Yu A, Somasundar P, Balsubramaniam A, Rose AT, Vona-Davis L, McFadden DW (2002). Vitamin E and the Y4 agonist BA-129 decrease prostate cancer growth and production of vascular endothelial growth factor. J Surg Res.

[26] Aerts E, Beckers S, Zegers D, Van Hoorenbeeck K, Massa G, Verrijken A, Verhulst SL, Van Gaal LF, Van Hul W (2016). CNV analysis and mutation screening indicate an important role for the NPY4R gene in human obesity. Obesity (Silver Spring).

[27] Misawa K, Kanazawa T, Misawa Y, Imai A, Uehara T, Mochizuki D, Endo S, Takahashi G, Mineta H (2013). Frequent promoter hypermethylation of tachykinin-1 and tachykinin receptor type 1 is a potential biomarker for head and neck cancer. J Cancer Res Clin Oncol.

[28] Misawa K, Misawa Y, Kondo H, Mochizuki D, Imai A, Fukushima H, Uehara T, Kanazawa T, Mineta H (2015). Aberrant methylation inactivates somatostatin and somatostatin receptor type 1 in head and neck squamous cell carcinoma. PLoS One.

[29] Misawa K, Mochizuki D, Endo S, Mima M, Misawa Y, Imai A, Shinmura K, Kanazawa T, Carey TE, Mineta H (2017). Site-specific methylation patterns of the GAL and GALR1/2 genes in head and neck cancer: potential utility as biomarkers for prognosis. Mol Carcinog.

[30] Lappano R, Maggiolini M (2011). G protein-coupled receptors: novel targets for drug discovery in cancer. Nat Rev Drug Discov.

[31] Wang Z (2016). Transactivation of epidermal growth factor receptor by G protein-coupled receptors: recent progress, challenges and future research. Int J Mol Sci.

[32] Chaplin R, Thach L, Hollenberg MD, Cao Y, Little PJ, Kamato D (2017). Insights into cellular signalling by G protein coupled receptor transactivation of cell surface protein kinase receptors. J Cell Commun Signal.

[33] Misawa K, Mochizuki D, Imai A, Endo S, Mima M, Misawa Y, Kanazawa T, Carey TE, Mineta H (2016). Prognostic value of aberrant promoter hypermethylation of tumor-related genes in early-stage head and neck cancer. Oncotarget.

[34] Misawa Y, Misawa K, Kanazawa T, Uehara T, Endo S, Mochizuki D, Yamatodani T, Carey TE, Mineta H (2014). Tumor suppressor activity and inactivation of galanin receptor type 2 by aberrant promoter methylation in head and neck cancer. Cancer.

[35] Huang WY, Hsu SD, Huang HY, Sun YM, Chou CH, Weng SL, Huang HD (2015). MethHC: a database of DNA methylation and gene expression in human cancer. Nucleic Acids Res.

[36] Gao J, Aksoy BA, Dogrusoz U, Dresdner G, Gross B, Sumer SO, Sun Y, Jacobsen A, Sinha R, Larsson E, Cerami E, Sander C, Schultz N (2013). Integrative analysis of complex cancer genomics and clinical profiles using the cBioPortal. Sci Signal.

[37] Toyooka S, Maruyama R, Toyooka KO, McLerran D, Feng Z, Fukuyama Y, Virmani AK, Zochbauer-Muller S, Tsukuda K, Sugio K, Shimizu N, Shimizu K, Lee H (2003). Smoke exposure, histologic type and geography-related differences in the methylation profiles of non-small cell lung cancer. Int J Cancer.

[38] Gu J, Berman D, Lu C, Wistuba II, Roth JA, Frazier M, Spitz MR, Wu X (2006). Aberrant promoter methylation profile and association with survival in patients with non-small cell lung cancer. Clin Cancer Res.

[39] Katz MH (2011). Multivariable Analysis: A Practical Guide for Clinicians and Public Health Researchers.

