# Supplementary material for: Genes encoding neuropeptide receptors are epigenetic markers in patients with head and neck cancer: a site-specific analysis
Source: Oncotarget. 2017 Jul 18;8(44):76318–28. doi: 10.18632/oncotarget.19356 (PMC5652708; doi:10.18632/oncotarget.19356)
Supplement: Supplementary file 1 [file oncotarget-08-76318-s001.pdf]

Genes encoding neuropeptide receptors are epigenetic markers in patients with head and neck cancer: a site-specific analysis

SUPPLEMENTARY MATERIALS

Supplementary Table 1: Methylation status of individual genes and associations with disease-free survival using Cox proportional hazards model in 216 patients

Gene	Adjusted HR (95% CI) †	P values
NPFFR1 methylation Yes vs No	0.767 (0.445-1.322)	0.34
NPFFR2 methylation Yes vs No	0.914 (0.505-1.653)	0.765
HCRTR1 methylation Yes vs No	1.530 (0.895-2.615)	0.120
HCRTR2 methylation Yes vs No	0.746 (0.446-1.248)	0.264
NPY1R methylation Yes vs No	1.643 (1.017-2.652)	0.042*
NPY2R methylation Yes vs No	1.981 (1.236-3.176)	0.005*
NPY4R methylation Yes vs No	1.145 (0.705-1.860)	0.585
NPY5R methylation Yes vs No	1.003 (0.616-1.635)	0.989

† Adjusted for mode of therapy, HPV status, smoking status, alcohol exposure and stage.

* $P<0.05$.

Supplementary Table 2: Patient and clinical characteristics

See Supplementary File 2

Supplementary Table 3: Real time MSP primer list

See Supplementary File 3

Supplementary Table 4: Q-RT PCR primer list

See Supplementary File 4

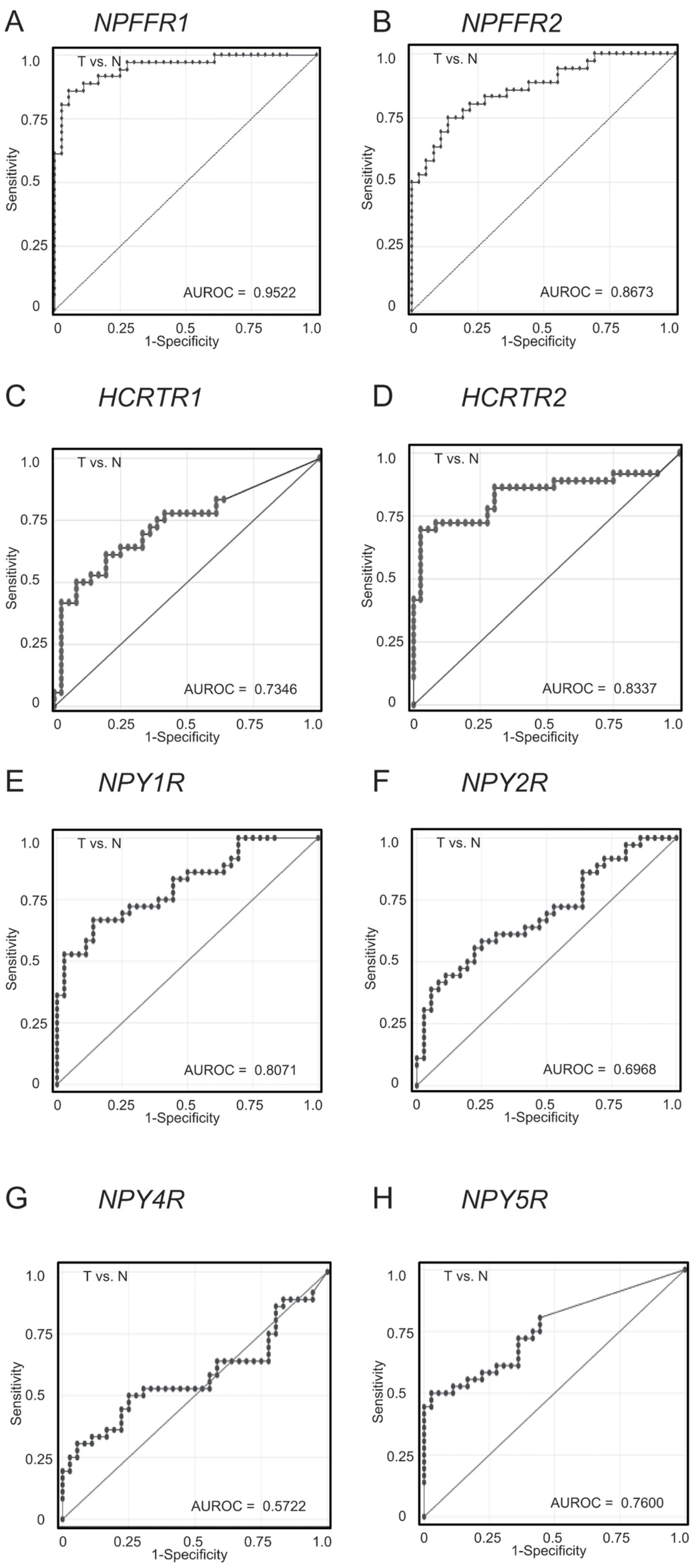

Supplementary Figure 1: Receiver operating characteristic (ROC) curves for the methylation markers in head and neck carcinomas versus adjacent normal mucosal tissue. Based on the ROC curve analysis, the sensitivity, specificity, and cutoff levels were 86.1%, 94.4%, and 0.0026 for *NPFFR1* **(A)**; 75.0%, 86.1%, and 0.082 for *NPFFR2* **(B)**; 61.1%, 80.6%, and 0.104 for *HCRTR1* **(C)**; 69.4%, 97.2%, and 0.099 for *HCRTR2* **(D)**; 44.4%, 91.7%, and 0.024 for *NPY1R* **(E)**; 94.4%, 13.9%, and 0.16 for *NPY2R* **(F)**; 86.1%, 19.4%, and 0.30 for *NPY4R* **(G)**; and 61.1%, 47.2%, and 0.025 for *NPY5R* **(H)**, respectively. T: tumor tissue, N: normal tissue.

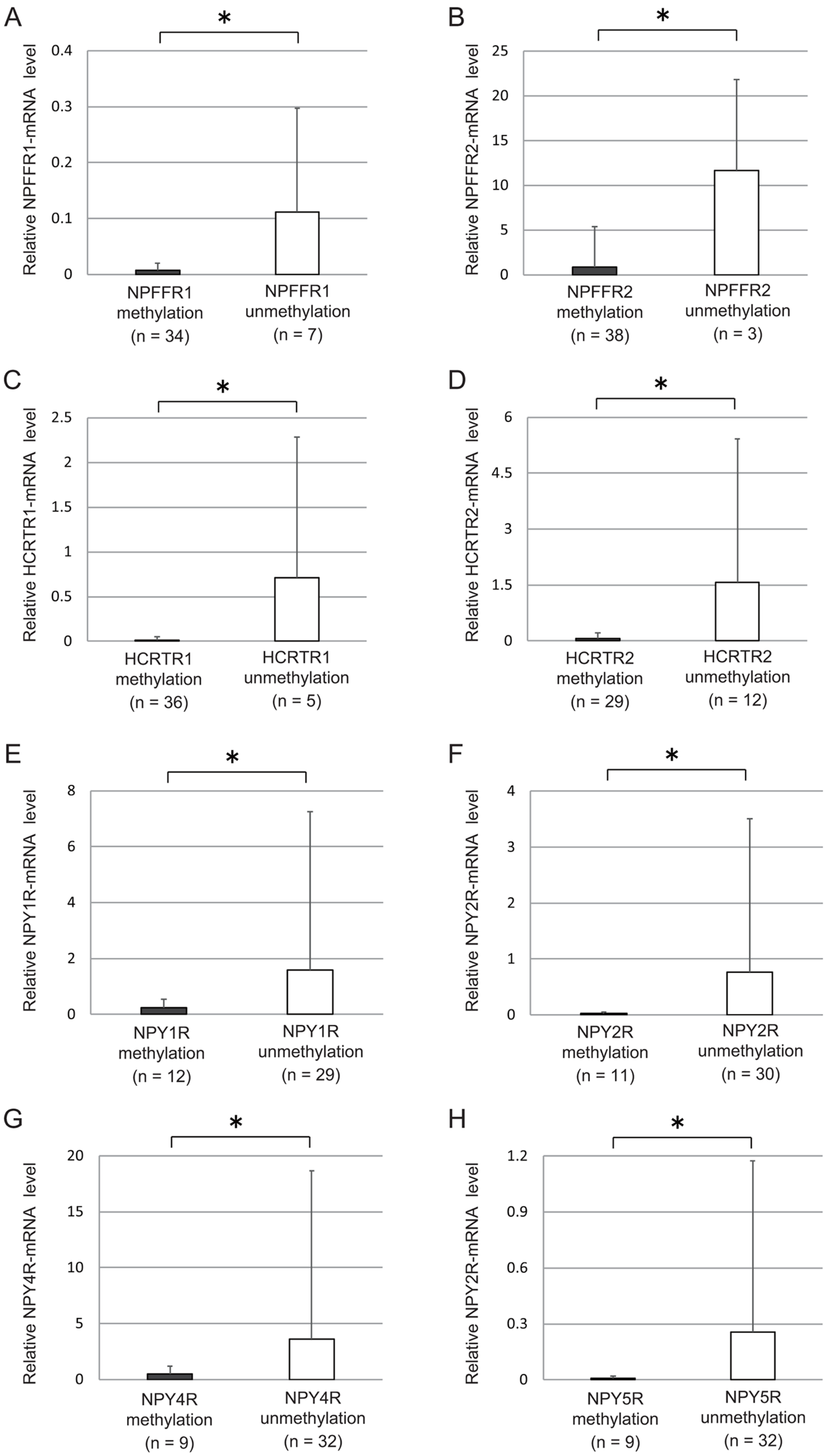

Supplementary Figure 2: mRNA levels for the eight neuropeptide receptor genes in 41 patients according to methylation status. The changes were significant as determined by Student's t-test. *P < 0.05.

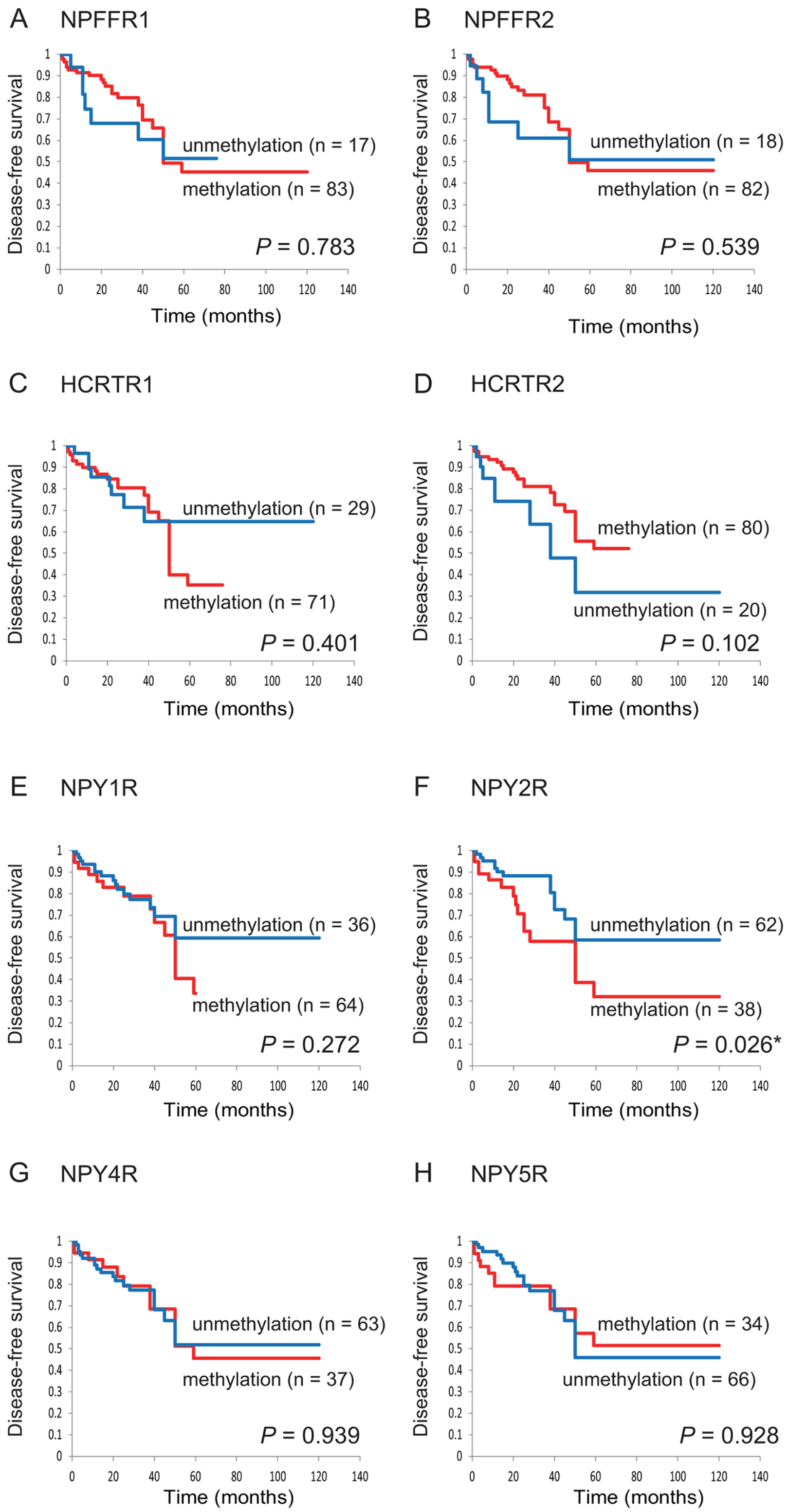

Supplementary Figure 3: Kaplan-Meier survival curves for the 100 patients without lymph node metastasis. Disease-free survival for **(A)** NPFFR1, **(B)** NPFFR2, **(C)** HCRTR1, **(D)** HCRTR2, **(E)** NPY1R, **(F)** NPY2R, **(G)** NPY4R and **(H)** NPY5R. The log-rank test was used to compare the survival times in patients with methylated (red lines) and unmethylated (blue lines) genes.

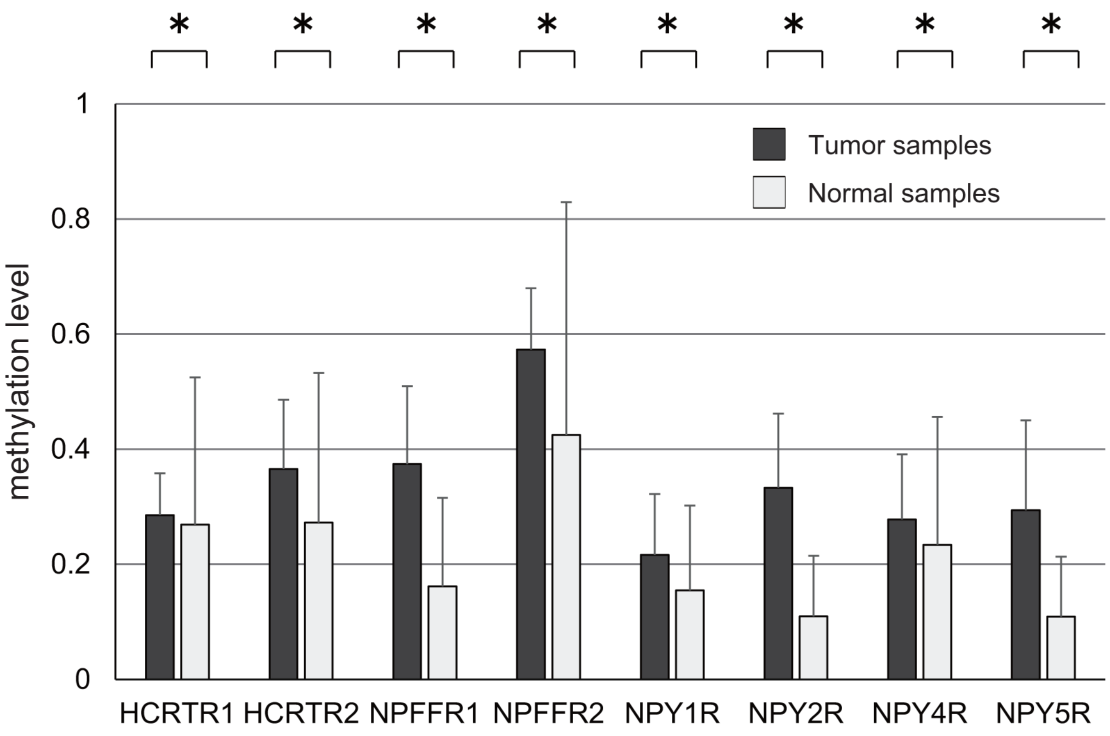

Supplementary Figure 4: Methylation status of the eight neuropeptide receptor genes in HNSCC and normal samples in TCGA database. The methylation data for *NPFFR1*, *NPFFR2*, *HCRTR1*, *HCRTR2*, *NPY1R*, *NPY2R*, *NPY4R*, and *NPY5R* in HNSCC and normal samples were collected from TCGA database. *$P < 0.005$.

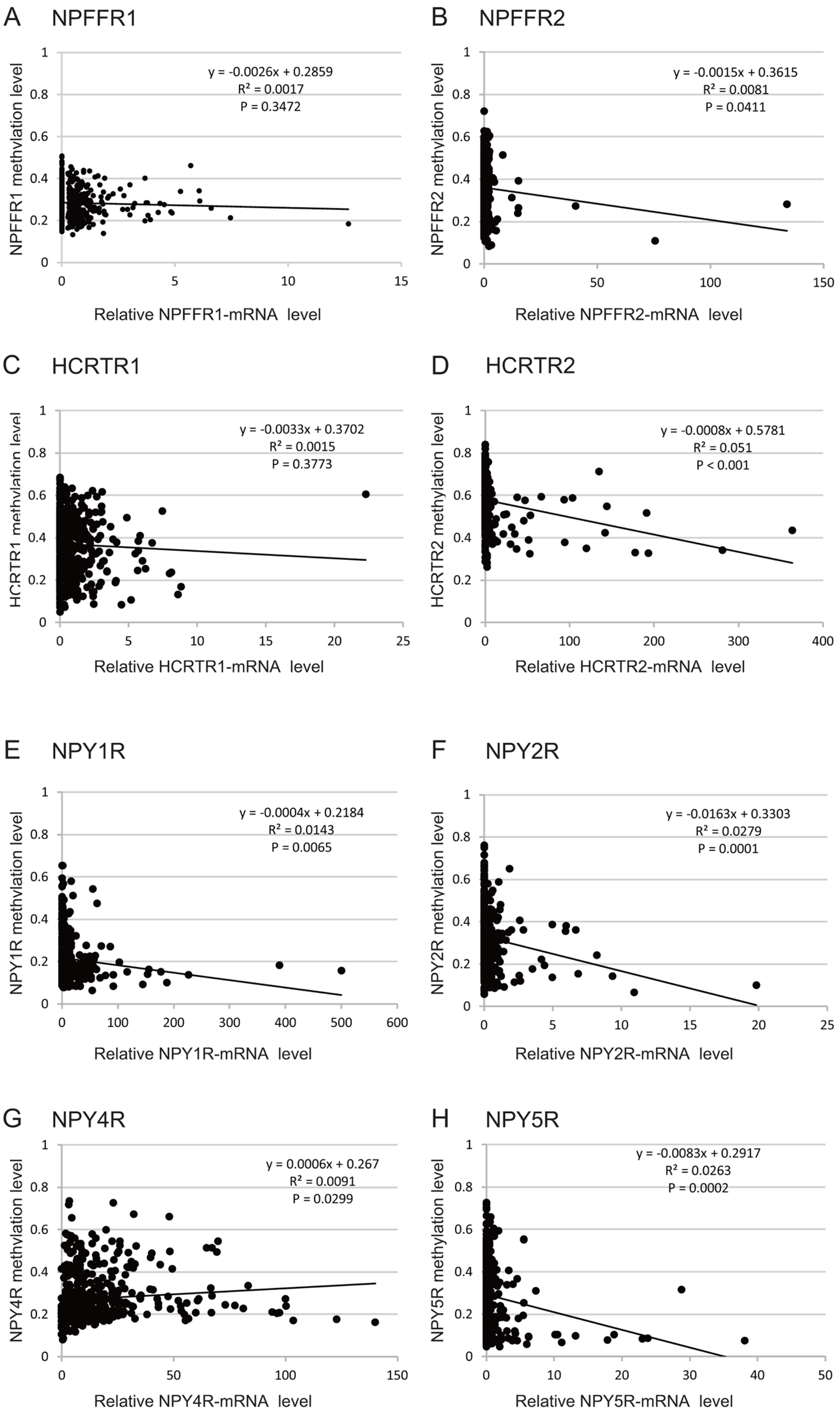

Supplementary Figure 5: Methylation and expression status of the eight neuropeptide receptor genes in HNSCCs in TCGA database. Scatter plot analysis for **(A)** *NPFFR1*, **(B)** *NPFFR2*, **(C)** *HCRTR1*, **(D)** *HCRTR2*, **(E)** *NPY1R*, **(F)** *NPY2R*, **(G)** *NPY4R*, and **(H)** *NPY5R*. Spearman rank correlation coefficient (R^2) and P values are shown.

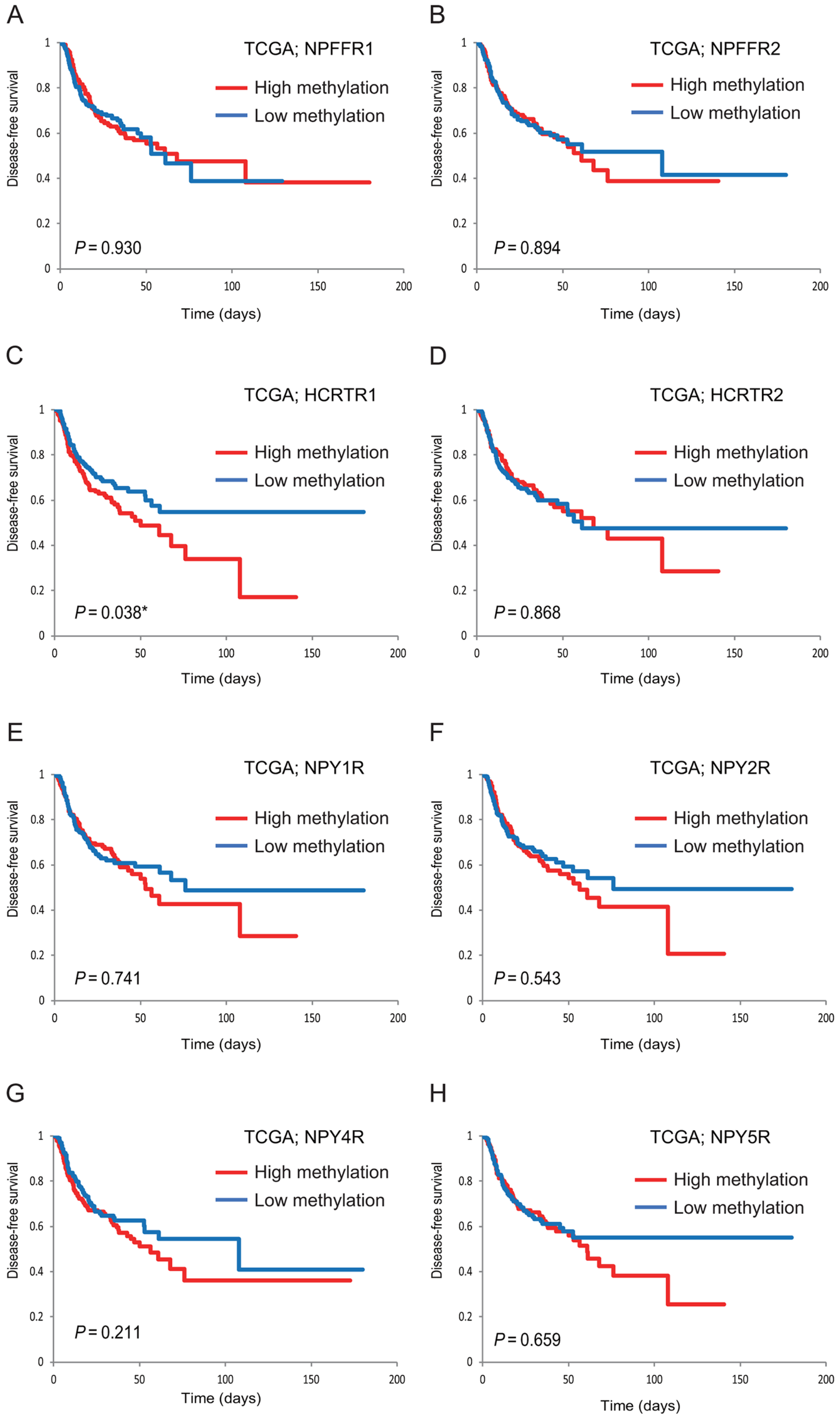

Supplementary Figure 6: Disease-free survival curves in TCGA cohort using median methylation as the cutoff. Disease-free survival curves for **(A)** *NPFFR1*, **(B)** *NPFFR2*, **(C)** *HCRTR1*, **(D)** *HCRTR2*, **(E)** *NPY1R*, **(F)** *NPY2R*, **(G)** *NPY4R*, and **(H)** *NPY5R*. The patients were divided into two groups.
