# Supplementary material for: Genes encoding neuropeptide receptors are epigenetic markers in patients with head and neck cancer: a site-specific analysis
Source: Oncotarget. 2017 Jul 18;8(44):76318–28. doi: 10.18632/oncotarget.19356 (PMC5652708; doi:10.18632/oncotarget.19356)
Supplement: Supplementary file 2 [file oncotarget-08-76318-s002.docx]

| **Supplementary Table 2: Patient and clinical characteristics** | | |
| --- | --- | --- |
| **Characteristic** | | **No. of patients (%)** |
| Age (years) | |  |
|  | < 70 | 149 (64.5) |
|  | ≥70 | 82 (35.5) |
| Gender | |  |
|  | Female | 36 (15.6) |
|  | male | 195 (84.4) |
| Smoking status | |  |
|  | smoker | 175 (75.8) |
|  | non smoker | 56 (24.2) |
| Alcohol exposure | |  |
|  | drinker | 169 (73.2) |
|  | non drinker | 62 (26.8) |
| Tumor site | |  |
|  | hypopharynx | 59 (25.5) |
|  | larynx | 45 (19.5) |
|  | oropharynx | 58 (25.1) |
|  | oral cavity | 69 (29.9) |
| Tumor size | |  |
|  | T1 | 27 (11.7) |
|  | T2 | 85 (36.8) |
|  | T3 | 43 (18.6) |
|  | T4 | 76 (32.9) |
| Lympho-node status | |  |
|  | N0 | 100 (43.3) |
|  | N+ | 131 (56.7) |
| Stage | |  |
|  | I | 20 (8.7) |
|  | II | 41 (17.7) |
|  | III | 42 (18.2) |
|  | IV | 128 (55.4) |
| HPV status | |  |
|  | positive | 37 (16.0) |
|  | negative | 192 (83.1) |
|  | n/a | 2 (0.9) |
| Treatment | |  |
|  | Surgery | 130 (56.3) |
|  | Surgery-RT±Chemo | 88 (30.1) |
|  | n/a | 13 (5.6) |
| Recurrence events | |  |
|  | positive | 85 (36.8) |
|  | negative | 146 (63.2) |
| n/a: no available information. | | |
| RT±Chemo: Radiation therapy ±Chemotherapy | | |
