# Supplementary material for: Genes encoding neuropeptide receptors are epigenetic markers in patients with head and neck cancer: a site-specific analysis
Source: Oncotarget. 2017 Jul 18;8(44):76318–28. doi: 10.18632/oncotarget.19356 (PMC5652708; doi:10.18632/oncotarget.19356)
Supplement: Supplementary file 3 [file oncotarget-08-76318-s003.docx]

**Supplementary Table 3: Real time MSP primer list**

**Gene**

NPFFR1

NPFFR2

HCRTR1

HCRTR2

NPY1R

NPY2R

NPY4R

NPY5R

ACTB

**Forward/Reverse**

F

R

F

R

F

R

F

R

F

R

F

R

F

R

F

R

F

R

**Sequence**

CGTAATTAAGTGTTCGGAGATCG

CCCAATTACGAACTCCGACGA

CGGCGGGTTAGTTTGGAGCG

CAACCGACGTCTATCCCCCG

CGGGTTTCGGGGTTGGAAGATA

CGGGTTTCGGGGTTGGAAGATA

CGCGTAGTTTTTTTTATCGTAA

CGAACATCACGAACTCAAATCCG

TTCGGGTTCGGGTTAGCGTTG

AACGAATCTCTAACGAAACCG CGAGTGAGTGCGGTGTTTAGGCG

CGAACGAACAACCGAAACAATC

AGGTTGGGCGGGCGTAGGCGGGA

CGAAACAAAACCGCGCCTACTT

AGTTACGTGTTTTCGAGACGT

CGTCCTACACCCCGACGATAA

TGGTGATGGAGGAGGTTTAGTAAGT

AACCAATAAAACCTACTCCTCCCTTAA

**Length (bp)**

94

67

107

98

92

101

101

103

133
