# Supplementary material for: Genes encoding neuropeptide receptors are epigenetic markers in patients with head and neck cancer: a site-specific analysis
Source: Oncotarget. 2017 Jul 18;8(44):76318–28. doi: 10.18632/oncotarget.19356 (PMC5652708; doi:10.18632/oncotarget.19356)
Supplement: Supplementary file 4 [file oncotarget-08-76318-s004.docx]

**Supplementary Table 4: Q-RT PCR primer list**

**Gene**

NPFFR1

NPFFR2

HCRTR1

HCRTR2

NPY1R

NPY2R

NPY4R

NPY5R

GAPDH

**Forward/Reverse**

F

R

F

R

F

R

F

R

F

R

F

R

F

R

F

R

F

R

**Sequence**

ACCTGCTGGTGGGCATCTTC

CTCATCTTGCATGTGGCATTGTC

CTCATCTTGCATGTGGCATTGTC

CAGATGGAGACATAATGGTGATGG

GAAGCTGTCTACGCCTGCTTCAC

CCTTAAACTGCTCCCGGAATTTG

TTGGGATGTTTGCCCATACTGA

TGAACTCCAAGGCAACAGCAAG

CATGTCCACGATGCACACAGA

GTCATCCGGGACCATAGGCTATAA

GGAAACGATTGCCAACTATACGA

GGCCCACTGAGTGTTGAGGA

GTGATGGTCTTCATCGTCACTTC

AAGCAGGTTGGTCACGTTGG

GCATTGCTGAGCAGCAGGTATTTA

TCTTTGTTGGACAATCCACAGCTTA

GCACCGTCAAGGCTGAGAAC

TGGTGAAGACGCCAGTGGA

**Length (bp)**

91

187

112

172

133

108

120

170

138
